# Understanding the Appropriate and Beneficial Use of Before and After Photos in Breast Surgery: A North American Survey

**DOI:** 10.1177/22925503221146783

**Published:** 2023-01-03

**Authors:** Shaishav Datta, Chantal R. Valiquette, Ron Somogyi

**Affiliations:** 1 Temerty Faculty of Medicine, 7938 University of Toronto, Toronto, ON, Canada; 2 Division of Plastic, Reconstructive & Aesthetic Surgery, Department of Surgery, 7938 University of Toronto, Toronto, ON, Canada

**Keywords:** before and after, breast reconstruction, breast surgery, crowdsourcing, informed consent, patient-centered care, patient photos, avant-après, chirurgie mammaire, consentement éclairé, photos des patients, production participative, reconstruction mammaire, soins axés sur les patients

## Abstract

**Introduction:** Before and after photographs (BAPs) in breast surgery have been identified as important components of the informed consent process. Currently, there is limited consensus on the contents and presentation of BAPs. This study collected the opinions of prior and prospective patients on this topic. **Methods:** A survey, based on criteria identified by our previous nominal group technique (NGT) study, was designed to obtain patient perspectives on BAPs in breast surgery. Amazon Mechanical Turk, a validated crowd-sourcing tool, was used to identify and survey a group of 72 participants who indicated that they had undergone or were planning to undergo breast surgery. Likert items were analyzed using either chi-squared or Fisher's exact test. **Results:** Most respondents were cis-gendered-women (89%), Caucasian (83%), and between 31 and 41 years old (38%). Respondents agreed that BAPs are important to the consent process, for enabling patient-centered care, and should be presented in standardized sets. BAPs should be more accessible through different platforms, display multiple time points to show the healing process, and have multiple views including close-ups of scars. Photos should be unaltered except for de-identification, and have more diversity with regard to patient gender, age, skin color, and body mass index. These results align with results from our NGT study. **Conclusion:** Through this study we have identified many criteria that BAPs should meet according to prior and prospective breast surgery patients. Surgeons should think critically about how they present BAPs during the consent process to ensure effective patient-centered care.

## Résumé

**Introduction :** En chirurgie mammaire, les photos avant-après (PAP) font partie des aspects importants du processus de consentement éclairé. À l’heure actuelle, le consensus sur le contenu et la présentation des PAP est limité. La présente étude visait à recueillir l’avis de patientes passées et prospectives sur le sujet. **Méthodologie :** Les chercheurs ont préparé un sondage reposant sur les critères qu’ils avaient établis lors de leur étude antérieure sur la technique du groupe nominal (TGN) pour obtenir les points de vue des patients sur les PAP en chirurgie mammaire. Ils ont utilisé l’outil de production participative validé MechanicalTurk d’Amazon pour repérer et sonder un groupe de 72 participants qui ont indiqué avoir subi ou planifié de subir une chirurgie mammaire. Ils ont analysé les énoncés de l’échelle de Likert au moyen du test du chi carré ou de la méthode exacte de Fisher. **Résultats :** La plupart des répondants étaient des femme cisgenres (89%), blanches (83%), âgées de 31 à 41 ans (38%). Ces répondants ont convenu que les PAP constituent un aspect important du processus de consentement, qu’elles favorisent des soins axés sur les patients et qu’elles doivent être présentées sous forme d’ensembles standardisés. Les PAP devraient être plus accessibles sur diverses plateformes, présenter divers moments du processus de guérison et de multiples points de vue, y compris des gros plans des cicatrices. Les photos devraient être inaltérées, à part pour la désidentification, et refléter une plus grande diversité de genres, d’âges, de couleurs de peau et d’indices de masse corporelle. Ces résultats concordent avec ceux de l’étude antérieure par la TGN. **Conclusion :** Grâce à la présente étude, les chercheurs ont relevé de nombreux critères que doivent respecter les PAP selon les patients passés et prospectifs en chirurgie mammaire. Les chirurgiens devraient recourir à la réflexion critique quant à la manière de présenter les PAP pendant le processus de consentement pour s’assurer de prodiguer des soins efficaces axés sur les patients.

## Introduction

Patient photos, specifically, before and after photographs (BAPs), have been identified as important aspects of patient education and surgical care.^1–5^ In particular, BAPs in breast surgery have been identified as important considerations for patients prior to deciding to undergo surgery.^6^ Given the landscape of social media, with widely available content that is often sensational rather than educational, it is important to evaluate what content is “appropriate” and “beneficial” for patients during surgical decision-making.^7–12^ There remains limited research into this area, and no consensus guidelines from governing bodies such as the American Society of Plastic Surgeons (ASPS), Canadian Society of Plastic Surgeons, and College of Physicians and Surgeons of Ontario.^13^ Recently, using Nominal Group Technique (NGT), we gathered eight surgeon experts to evaluate how BAPs may be used in the informed consent process for breast surgery.^13^ From this study, we established five themes that should be in a set of standardized BAPs for use in the informed consent process, which included: having a (1) clearly defined audience, (2) using patient photographs for displaying BAPs, (3) standardizing the anatomy that is represented, (4) displaying a range of patient outcomes, and (5) maintaining photographic integrity.^13^ The NGT study began the important discussion around the use of BAPs in breast surgery and their consideration as part of informed consent by exploring related literature and beginning the process of determining what categories should be included on this topic. While expert consensus was valuable, informed consent should also be evaluated by the patient's understanding of the information provided and by its utility and relevance to the procedure described. Little is known about what criteria patients find important when evaluating BAPs or how patients perceive the utility of BAPs when deciding on breast surgery. As the next step in understanding these concepts, investigators planned to expand the study through patient evaluation of BAPs in breast surgery.

In order to ensure the study could be as generalizable as possible and to expand the scope by including patients beyond single institutional centers, crowdsourcing was chosen as the methodology for collection of survey data. Crowdsourcing has been extensively used in the last decade for a wide variety of application in health science and medicine research due to its ability to allow researchers to access large populations worldwide.^14^ Specifically, Amazon Mechanical Turk (MTurk) (Amazon, Seattle, Washington) a web-based tool, offers a fast and low-cost option to access a large and generalizable population of survey-takers.^15–18^

The primary objectives of this study were to (1) evaluate the sentiments of previous and prospective breast surgery patients regarding the use of BAPs in breast surgery; (2) compare these results to our previous NGT study that examined responses from surgeon experts; (3) better define criteria that can be used to build consensus on appropriate and beneficial use of BAPs in breast surgery. Ultimately, we aim to provide guidelines for clinicians that outline how to use BAPs in breast surgery to ensure better and more informed patient-centered care.

## Methods

**Figure 1.**
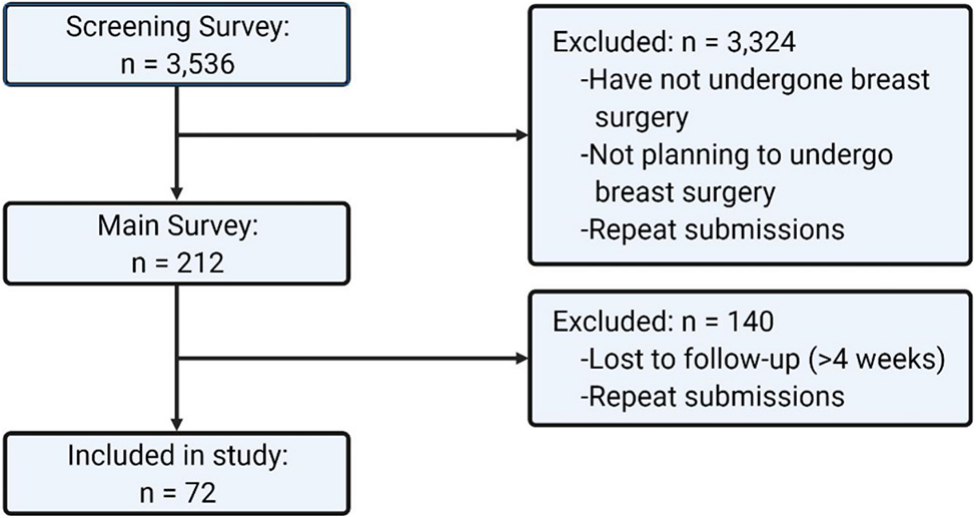
Eligibility and recruitment of participants flowchart. Created with BioRender.com.

Using MTurk, the sentiments and opinions of a general population (>18 years of age) in Canada and the United States of America (USA) regarding the use of BAPs in breast surgery were collected and assessed (Figure 1). All respondents agreed to an electronic consent form prior to participation. The study procedures were approved by the Regional Hospital Research Ethics Board (REB number 21-0034).

Participants were recruited via a two-stage process. The screening survey was an open survey, available to all MTurk workers above the age of 18 years old and living in Canada or the USA. The screening survey collected respondent characteristics such as gender, race, age, highest level of education, province/territory/state of residence, annual household income, and whether or not they planned to undergo or had undergone breast surgery. This survey was administered between July 12, 2021, and August 9, 2021. The screening survey was closed after no new responses were collected for more than 1 week (*n* = 3536) (Supplemental File 1). Respondents who indicated that they planned to undergo or had undergone breast surgery were then recruited to complete the main survey (*n* = 212). The main survey assessed participant's opinion on the utility, content, and presentation of BAPs in breast surgery through Likert-item questions and short answer questions and was administered between October 10, 2021, and December 10, 2021 (Supplemental File 2). The main survey was closed after no new responses were collected for more than 1 month (*n* = 72).

Chi-square tests were used to compare the proportions of categorical variables for Likert-type questions indicating agreement (agree or strongly agree) versus those indicating disagreement (disagree or strongly disagree). Respondents indicating neither agreement nor disagreement were excluded from analysis. Fisher's exact test was used to compare categorical variables that had an expected frequency of less than five in a particular cell.

## Results

### Demographics

**Figure 2.**
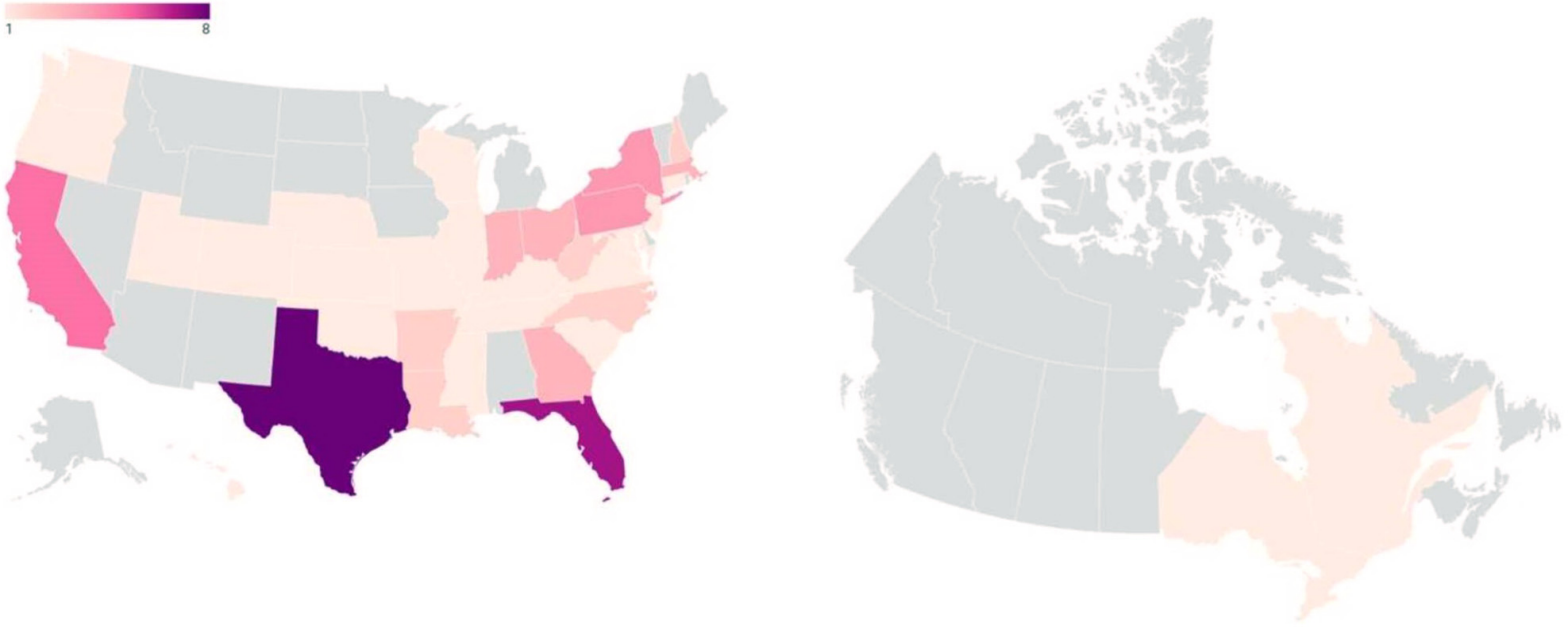
State/province/territory of residence of survey participants (USA: *n* = 70, Canada: *n* = 2).^19^

A total of 72 participants who completed the main survey were included in the study. Demographic analysis showed majority of respondents were cis-gendered women (89%) (Table 1), Caucasian (83%) (Table 1), and between the ages of 31 and 41 years old (38%) (Table 1). The breakdown of participants’ annual reported income and levels of education are represented in Table 1. Most respondents were from the USA (97%) (Figure 2).

**Table 1.** Gender Identity^a^, Age, Racial Background^b^, Highest Level of Education^c^, and Reported Annual Household Income of Survey Participants.

	Number of respondents
*Gender*	
Woman (cis gender)	64
Man (cis gender)	4
Transgender and Gender Diverse	4
*Age (years)*	
18–20	0
21–30	15
31–40	27
41–50	13
51–60	14
Older than 60	3
*Racial background*	
Caucasian	57
Black	7
Hispanic	3
Indigenous (North American)	2
East Asian	1
South Asian	1
Central Asian	1
Other	0
*Highest level of education*	
High school or equivalent	4
Trade/technical/vocational training	11
Bachelor's degree	26
Some college credit, no degree	13
Master's degree	13
Doctorate degree	3
Professional degree	2
Other	0
*Individual income*	
Prefer not to say	1
<$25 000	8
$25 000–$50 000	20
$50 000–$100 000	23
$100 000–$200 000	16
>$200 000	4
*Breast surgery*	
Aesthetic – undergone	30
Aesthetic – planning to undergo	37
Reconstructive – undergone	14
Reconstructive – planning to undergo	7

^a^ “Transgender and Gender Diverse” includes responses: Woman (transgender), Man (transgender), Two spirit indigenous person, gender diverse, and unsure.

^b^ “Other” includes responses: Indigenous person (not from North America), Middle Eastern/North African, Southeast Asian, West Asian, and Prefer not to disclose.

^c^ “Other” includes responses: No schooling completed, nursery school to eighth grade, and some high school (no diploma).

### BAP Survey

Overall, 93% of respondents reported that they had undergone or were planning to undergo aesthetic breast surgery, 29% of respondents reported that they had undergone or were planning to undergo reconstructive breast surgery, and 22% indicated that they had undergone or were planning to undergo both. The two most utilized resources for patient education prior to consenting to surgery were physician expertise (60%) and advice from other patients who had undergone similar surgeries (60%). The next three most utilized resources were websites run by medical groups/organizations/governing bodies (26%), accredited plastic surgeon's social media (24%), and other influencers (24%).

**Figure 3.**
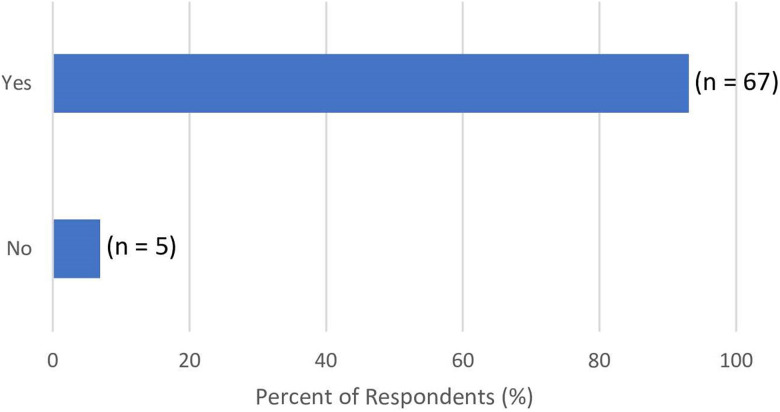
Response to “Have you ever looked at another patient's BAPs similar to your surgery(ies) or potential surgery(ies)?”.

Most respondents reported having viewed another patient's BAPs similar to their surgery(ies)/potential surgery(ies) (92%) (Figure 3). Of these, 70% stated their reason for viewing BAPs was to set expectations related to specific procedures and/or scarring outcomes, while 30% stated they were to assess the surgeon's abilities. Among those who viewed BAPs, 95% of respondents reported feeling more empowered/confident consenting to surgery after viewing BAPs. The remaining 5% responded “No,” with comments suggesting that BAPs did not reduce their confidence in consenting to their surgery, but rather reduced their confidence in a particular surgeon's ability to perform their surgery.

There was agreement among survey respondents regarding the importance of BAPs in the consent process, with 76% respondents agreeing that they felt BAPs are an accurate representation of what to expect from breast surgery (Table 2A). Although respondents agreed that both physician and non-physician provided BAPs are widely available, there should be more access to both types of resources (Table 2B). Respondents agreed that physician-provided BAPs are more helpful than those found in other resources (Table 2B). Survey respondents suggested that the intended audience for breast surgery-related BAPs were patients in doctor's offices and patients considering surgery, not the general public (Table 2C). There was no consensus reached on whether patient's families are the intended audience (Table 2C). Survey respondents indicated that anywhere, including their home or the doctor's office, served as an appropriate place for patients to view breast surgery-related BAPs (Table 2D). Websites, private links sent to patients, brochures/print media, and devices used at doctor's offices all represent appropriate platforms to show patients BAPs (Table 2E). In contrast, there was no consensus on whether social media represents an appropriate platform for this purpose (Table 2E). The key theme among respondents was ensuring that patients had consented to their BAPs being used on a particular platform before presenting it to other prospective patients. Respondents indicated that hand drawings were not an appropriate method of representing BAPs in breast surgery, but that unedited photographs, non-photoshopped lighting-edited photographs, videos, simulations, and in-person results from previous patients all represented appropriate forms of representing BAPs in breast surgery (Table 2F). There was no consensus reached on whether formal illustrations are appropriate for representing BAPs in breast surgery (Table 2F). Respondents indicated that a standard set of BAPs should include when the photographs were taken, associated documentation required for consent, patient testimonials, consistent pre- and post-operative lighting, equivalent size ratio between BAPs, multiple picture views (front, side, arms-up, and 45° profiles; and close-up of scars) with standard body positions, consistent patient exposure, and the same backgrounds (Table 2G). Additionally, respondents felt and agreed that there needs to be more diversity in skin tones and age of patients represented in BAPs in breast surgery (Table 2G). Respondents indicated that a set of standard BAPs should include pictures from immediately after the surgery and future dates including 2 weeks, 6 weeks, 3 months, 6 months, and 1 year after the surgery (Table 2H). The key theme among respondents was that BAPs should display the healing process at different points after the surgery. There was no consensus reached on the utility of photographs from during the surgery in a standard set of BAPs (Table 2H). Respondents were in agreement that blacking out/covering or using photoshop to edit out and de-identify BAPs is an acceptable practice (Table 2I). Further, they agree that patient identifiers, including name or location, should not be included in BAPs (Table 2I). With regards to labels that should be included in BAPs, respondents agree that age of patient, time from surgery, procedures performed (visible in photograph), information about implants, incision type, and diagnosis prior to treatment should be included (Table 2J). Respondents agree that the range of outcomes that should be represented across sets of BAPs include best results, average results, and achievable/reproducible results (Table 2K). There was no consensus reached on the use of different types of casual clothing in BAPs (Table 2L); however 62% of respondents felt that patient photos need to include some form of casual clothing with weak statistical significance. Most respondents (64%) were in favor of no limit on the number of photographs to be used in a standard set of BAPs, with 98.61% of respondents requiring at least three photos (Table 2M).

**Table 2.** Responses to the Main Survey Questionnaire.

Question	SD	D	N	A	SA	*P*-value
*Section A: Questions related to utility of BAPs in the consent process*						
BAPs are integral to the informed consent process	2	5	4	24	37	5.8 × 10^−11^
BAPs support the patient in contributing to the decision-making process	3	1	3	16	49	1.2 × 10^−9^
I would recommend that patients look at BAPs prior to consenting to surgery	4	0	2	10	56	1.5 × 10^−9^
BAPs are an accurate representation of what to expect from surgery	3	5	9	37	18	3.2 × 10^−9^
*Section B: Questions related to availability and accessibility of BAPs*						
Physician-provided BAPs are widely available	4	2	13	29	24	9.4 × 10^−10^
I believe there should be increased access to physician-provided BAPs	2	3	10	21	36	4.0 × 10^−11^
BAPs are widely available through “outside resources”	4	5	20	22	21	2.4 × 10^−6^
I believe there should be increased access to BAPs from “outside resources”	5	7	17	16	27	2.9 × 10^−5^
I believe physician-provided BAPs are more helpful than “outside resources”	5	8	17	15	27	9.2 × 10^−5^
I believe “outside resources” are equally as helpful or more helpful than physician-provided BAP	3	22	22	11	14	.67
*Section C: “The intended photographic audience of BAPs are:”*						
Office patients	2	3	11	26	30	6.6 × 10^−11^
Patients considering surgery	2	1	0	12	57	1.1 × 10^−10^
Patients’ families	8	18	21	19	6	.89
General public	15	23	17	13	4	.0046
General public excluding minors	14	19	24	9	6	.0094
*Section D: “Appropriate places for patients to view BAPs are:”*						
Anywhere	4	15	9	21	23	.0016
Anywhere on a secure site	0	6	7	25	34	4.9 × 10^−11^
Home	1	2	8	25	36	2.4 × 10^−9^
Doctor's office	3	9	5	21	34	1.5 × 10^−7^
*Section E: “Appropriate platforms to show patients BAPs are:”*						
Websites	1	7	7	33	24	1.2 × 10^−9^
Social media	16	15	12	17	12	.80
Private links sent to patients	0	4	2	17	49	1.5 × 10^−9^
Brochures/print media	6	9	10	31	16	4.8 × 10^−5^
Devices at the doctor's office	0	0	4	26	42	2.2 × 10^−13^
*Section F: “Appropriate forms of presenting BAPs are:”*						
Hand drawings	25	22	8	11	6	.00018
Formal illustrations	10	19	6	28	9	.32
Unedited photographs	1	1	2	22	46	2.7 × 10^−11^
Edited photographs – lighting only (not photoshopped)	4	6	7	23	32	2.4 × 10^−8^
Videos	3	2	2	28	37	7.4 × 10^−13^
Simulations (3D/morphed)	8	8	15	26	15	.00093
In-person results from previous patients	6	7	13	20	26	1.7 × 10^−5^
*Section G: “Standard clinical BAPs should include:”*						
When the photographs were taken	0	1	3	23	45	2.0 × 10^−12^
Documentation required for informed consent	6	6	18	13	29	4.5 × 10^−5^
Testimonials	4	4	11	22	31	8.3 × 10^−9^
Consistent lighting (pre- and post-operative)	0	2	0	28	42	1.2 × 10^−11^
1:1 size ratio between BAPs	1	2	6	17	46	1.1 × 10^−9^
Picture views: Front profile, side profile ±45° profile	1	3	1	27	40	5.6 × 10^−10^
Arms up view	2	1	15	22	32	2.0 × 10^−8^
Close-up of scars and deformities	2	2	6	30	32	6.6 × 10^−9^
Same body positions/standardized positions	0	2	2	27	41	2.7 × 10^−11^
Consistent patient exposure	0	2	8	25	37	3.1 × 10^−10^
Same backgrounds	0	4	12	26	30	6.2 × 10^−8^
Unaltered (no photoshop/filter)	0	2	2	17	51	2.7 × 10^−11^
More diversity (ie, range of skin tones)	10	7	12	12	31	.00079
*Section H: “Standard clinical BAPs should include pictures from:”*						
During the surgery	6	24	19	10	13	.34
Immediately after the surgery	3	9	10	23	27	1.4 × 10^−6^
2 weeks after the surgery	2	3	1	29	37	4.5 × 10^−13^
6 weeks after the surgery	0	0	1	28	43	3.5 × 10^−14^
3 months after the surgery	2	0	4	23	43	6.2 × 10^−11^
6 months after the surgery	1	2	4	20	45	5.0 × 10^−10^
1 year after the surgery	0	3	5	20	44	4.2 × 10^−10^
*Section I: “With regards to de-identifying patients, BAPs should have:”*						
Identifiers fuzzed out/blacked out/covered	2	1	9	16	44	2.2 × 10^−30^
Identifiers erased/“photoshopped” out	3	5	9	20	35	3.2 × 10^−9^
No patient identifiers	2	2	8	12	48	7.8 × 10^−32^
All digital tags (name, location)/file name removed	2	1	7	9	53	4.6 × 10^−34^
*Section J: “The following labels should be included in BAPs:”*						
Age of patient	2	5	6	29	30	1.6 × 10^−10^
Time from surgery	2	5	4	19	42	5.8 × 10^−11^
All procedures performed (visible in photograph)	0	3	4	24	41	2.4 × 10^−30^
All device information (ie, shape/size/position of implant)	2	0	6	31	33	1.2 × 10^−26^
Incision type	0	2	5	30	35	9.1 × 10^−28^
Diagnosis, previous treatments	0	4	9	22	37	1.9 × 10^−27^
*Section K: “The range of outcomes that should be represented in BAPs should include:”*						
Best results	0	4	10	21	37	3.1 × 10^−27^
Average results	0	2	4	13	53	4.2 × 10^−35^
Achievable/reproducible results	0	1	2	15	54	3.3 × 10^−36^
*Section L: “Casual clothing, such as the following, should be included in BAPs:”*						
Shirts without cleavage	10	17	22	17	6	.57
Shirts with cleavage	11	16	20	18	7	.78
Bathing suits	9	14	16	21	12	.18
Patient photos need to be shown in casual clothing	10	10	20	14	18	.096
*Section M: “I believe a representative set of before and after photographs should include:”*						
	Number of Respondents					
3 photographs	13					
10 photographs	12					
No limit on photographs	46					

*P*-values highlighted in red are >.01 (SD, strongly disagree; D, disagree; N, neither agree nor disagree; A, agree; SA, strongly agree).

## Discussion

In this study, previous and prospective breast surgery patients from Canada and the USA completed a survey assessing the use of BAPs in breast surgery to enable patient-centered care. The survey probed participant opinions on criterion items, established previously in our NGT study, related to content and presentation of BAPs.^13^

The main takeaways from our study were that BAPs should be standardized, have increased accessibility on various platforms, include multiple time points following surgery to show the healing process, and present multiple views that are unaltered except for de-identification. Surgeons are encouraged to have a representative set of BAPs that are catered towards a diverse set of patient profiles, which takes into consideration factors such as gender, skin-color, age, and body mass index. Similar to previously established literature, our study finds that previous and prospective patient consider BAPs in breast surgery important to the consent process and for enabling patient-centered care.^6^

Additionally, this study furthers the work of our previous NGT study as the findings are in consensus for main categories such as patients considering surgery being the intended photograph audience, use of photographic images for BAPs, defining the standard clinical photograph by having patients in the same body position, anonymizing images by removing all digital tags and patient identifiers, not limiting the number of photograph sets needed for sufficient representation, and representing a surgeon's average outcomes.^13^ Additionally, our findings strengthen previous literature that suggests BAPs are used in surgical decision-making, with most respondents (92%) reporting having viewed another patient's BAPs similar to their surgery/potential surgery.^6^ Similar to the NGT study, findings were also suggestive that BAPs should be a key part of the informed consent process, with 95% of respondents who viewed BAPs reporting feeling more empowered/confident consenting to surgery after viewing BAPs.^13^ An interesting difference to note was that the NGT study did not endorse discussion of a “Complications” category, but patient survey respondents specifically mention they looked at BAPs to set expectations related to specific procedures and/or scarring outcomes (70%) or assess the surgeon's abilities (30%) (Figure 3). Furthermore, while patients endorsed seeking out BAPs to assess for scarring, there was no mention of other acute (infection, hematoma, seroma, flap necrosis, etc) or chronic (hypertrophic or keloid scarring, capsular contracture, implant rupture, flap necrosis, fat herniation, abdominal hernia, etc) complications related to breast surgery. Given that patients are interested in having complications represented in BAPs, further investigation is warranted to reconcile patient and physician perspectives in future studies. This will help elucidate the role of BAPs in representing suboptimal outcomes and its effect on the informed consent process.

While surgeon experts endorsed websites as an appropriate platform for presenting BAPs, this study found patients also believe private links sent to patients, brochures/print media, and devices used at a doctor's office are appropriate (Table 3). The patient study also broadened ideas of how the images should be displayed, not only endorsing photographs, but also unedited photographs, non-photoshopped lighting-edited photographs, videos, simulations, and in-person results from previous patients (Table 3). Another important differentiator in this study was the patient recognition of the importance of diversity in photo sets including age, gender, body mass index, and skin tone (Table 3). It was also interesting to see the contrast regarding BAPs with patients wearing casual clothing; a criterion not endorsed in the NGT study, but 62% of patients indicating it may be relevant with weak statistical significance (Table 3). Overall, while many of the NGT findings are strengthened by this study, it does suggest that there are certain areas to further clarify and delineate when establishing comprehensive guidelines for appropriate use of BAPs in breast surgery.

**Table 3.** Consensus between Surgeon Experts and Patients.

Endorsed by both patients and surgeons	Endorsed by patients only
Endorsed statements	
BAPs are an important component of the informed consent process	Sets of BAPs should include patients with casual clothing (weak significance)
Endorsed criterion	
*Intended audience for BAPs*	
Patients considering surgery	Office patients
*Appropriate places for patients to view BAPs*	
Anywhere	
*Appropriate platforms to view BAPs*	
Websites	Private links Brochures/print media Devices in doctor's office
*Appropriate forms of presenting BAPs*	
Photographs	Videos Simulation In-person results
*Standard clinical BAPs should include*	
Consistent lighting 1:1 ratio Front profile and side profile views Same body positions Same background No digital altering/filters (except de-identification)	Date of photo taken Documentation required for consent Patient testimonials Arms up view Close up of scars
*Standard clinical BAPs should include pictures from*	
1 year after the surgery	Immediately after the surgery 2 weeks after the surgery 6 weeks after the surgery 3 months after the surgery 6 months after the surgery
*BAPs should be anonymized by*	
Removing patient identifiers Removing digital tags	Fuzzing out/blacking out/covering up Photoshopping “out”
*Labels to be included in BAPs*	
Time from surgery	Age of patient Procedures performed Device/implant information Incision type Diagnosis and previous treatments
*Range of outcomes to be represented*	
Average results Achievable/reproducible results	Best results

Note There Were No Items Endorsed by the Surgeon Expert Group Only.

With regards to generalizability of respondent characteristics, our survey participants reflect the general demographics of a patient seeking breast surgery in the USA (Table 1).^20^ As per the ASPS statistics 2020, most patients who had undergone breast surgery were female (95%) and aged 30–39 (30%).^20^ Additionally, most patients who had undergone any cosmetic breast procedure were Caucasian, which aligned with our patient population (Table 1).^20^

A limitation of our study is our small sample size. However, our study employed a rigorous pre-screening survey, which yielded us high specificity with regards to the specific characteristics of our survey takers, reflecting that of a previous or prospective breast surgery patient. In this way, each participant has high information power.^21^ Another limitation of our study is that as per the crowdsourcing nature of the platform, MTurk workers are compensated for completion of surveys, and thus the survey participants are subject to self-selection bias. MTurk workers can choose to complete surveys based on amount of compensation, familiarity with type of task, interest in the nature of the task, and/or reviews from other participants who have completed the task.^22,23^ Even when workers start a survey, they are not required to complete the task if they find that the payment does not seem adequate for the time spent, if the task is overly complex, or if the task is uninteresting.^22^ Given our survey time had an average completion time of 24 min, it is possible that the length of the survey was a deterrent for some participants. Future work should aim to optimize the balance between survey length and participant response rate in order to gather as much information as possible. This sheds some light as to why our main survey had 66% nonparticipation. Additionally, the average half-life of an MTurk worker is approximately 1-year.^15^ Our study spanned 5 months, and as such, it is possible that this contributed to the high nonparticipation rate in our main survey. With regards to the disparity in proportion of participants from Canada and the USA, our participant population reflects the worldwide MTurk worker population of Americans (75%) to Canadians (1%) (Figure 2).^15^

## Conclusion

We provide a patient perspective on the content and presentation of breast surgery BAPs intended for consumption by prospective patients. The “appropriate” use of BAPs in breast surgery is important to the informed consent process and for patient-centered care, and thus surgeons should critically evaluate how to present BAPs in their practice. Additionally, this study demonstrates the utility of Amazon's MTurk as a useful tool for gathering patient perspectives in medical research. Moving forward, we plan to use these findings, along with those from our previous NGT study, to implement a Nation-wide Delphi study examination, which will serve as the basis for establishment of recommended guidelines for the “appropriate” and “beneficial” use of BAPs in breast surgery.

## Supplemental Material

sj-docx-1-psg-10.1177_22925503221146783 - Supplemental material for Understanding the Appropriate and Beneficial Use of Before and After Photos in Breast Surgery: A North American Survey Supplemental material, sj-docx-1-psg-10.1177_22925503221146783 for Understanding the Appropriate and Beneficial Use of Before and After Photos in Breast Surgery: A North American Survey by Shaishav Datta, Chantal R. Valiquette and Ron Somogyi in Plastic Surgery

sj-docx-2-psg-10.1177_22925503221146783 - Supplemental material for Understanding the Appropriate and Beneficial Use of Before and After Photos in Breast Surgery: A North American Survey Supplemental material, sj-docx-2-psg-10.1177_22925503221146783 for Understanding the Appropriate and Beneficial Use of Before and After Photos in Breast Surgery: A North American Survey by Shaishav Datta, Chantal R. Valiquette and Ron Somogyi in Plastic Surgery
