# Supplementary material for: Understanding the Appropriate and Beneficial Use of Before and After Photos in Breast Surgery: A North American Survey
Source: Plast Surg (Oakv). 2023 Jan 3;32(3):404–12. doi: 10.1177/22925503221146783 (PMC11298139; doi:10.1177/22925503221146783)
Supplement: sj-docx-1-psg-10.1177_22925503221146783 - Supplemental material for Understanding the Appropriate and Beneficial Use of Before and After Photos in Breast Surgery: A North American Survey [file sj-docx-1-psg-10.1177_22925503221146783.docx]

**Supplement 1. Screening Survey Questions**

1. What gender category do you identify with (select all that may apply)?
   1. Men (Cisgender where biological sex aligns with gender identity)
   2. Men (Transgender where biological sex does not align with gender identity)
   3. Women (Cisgender where biological sex aligns with gender identity)
   4. Women (Transgender where biological sex does not align with gender identity)
   5. Two Spirit Indigenous person
   6. Gender Diverse
   7. Unsure
   8. Prefer not to disclose
2. Do you identify with any of the following racial groups?
   1. Black (e.g. African, Caribbean, Canadian, etc.)
   2. Central Asian (e.g. Kazakh, Afghan, Tajik, Uzbek, Caucasus, etc.)
   3. East Asian (e.g. Chinese, Japanese, Korean, etc.)
   4. Indigenous person - from North America
   5. Indigenous person - from another country
   6. Latina/Latino/Latinx/Hispanic
   7. Middle Eastern or North African (e.g. Egyptian, Saudi, Moroccan, etc.)
   8. South Asian (e.g. Indian, Pakistani, Sri Lankan, East Indian from Guyana, Indo-Caribbean, etc.)
   9. Southeast Asian (e.g. Cambodian, Indonesian, Laotian, Vietnamese, Thai, etc.)
   10. West Asian (e.g. Armenian, Turkish, Persian, etc.)
   11. White (e.g. Southern European, Eastern European)
   12. Prefer not to disclose
3. What is your age?
   1. 18-20
   2. 21-30
   3. 31-40
   4. 41-50
   5. 51-60
   6. Older than 60
4. What is the highest degree or level of school you have completed?
   1. No schooling completed
   2. Nursery school to 8^th^ grade
   3. Some high school, no diploma
   4. Some college credit, no degree
   5. Trade/technical/vocational training
   6. Bachelor’s degree
   7. Master’s degree
   8. Professional degree
   9. Doctorate degree
5. What is your annual household income?
   1. Less than $25,000
   2. $25,000 - $50,000
   3. $50,000 - $100,000
   4. $100,000 - $200,000
   5. More than $200,000
   6. Prefer not to say
6. What province/territory/state do you reside in?
   1. Canada: ___________
   2. United States: ___________
7. Have you had any aesthetic/cosmetic surgery?
   1. Yes
   2. Planning to undergo in <6 months
   3. Planning to undergo at some point in the future
   4. Considering undergoing
   5. No
8. If you answered "Yes", "Planning to undergo", or "Considering undergoing", please specify the specific surgery below (eg. facelift/rhytidectomy). If you answered "No", please type N/A.

______________________________________________

1. Have you had reconstructive surgery?
   1. Yes
   2. Planning to undergo in <6 months
   3. Planning to undergo at some point in the future
   4. Considering undergoing
   5. No
2. If you answered "Yes", "Planning to undergo", or "Considering undergoing", please specify the specific surgery below (eg. tendon repair). If you answered "No", please type N/A.

______________________________________________
