# Supplementary material for: Understanding the Appropriate and Beneficial Use of Before and After Photos in Breast Surgery: A North American Survey
Source: Plast Surg (Oakv). 2023 Jan 3;32(3):404–12. doi: 10.1177/22925503221146783 (PMC11298139; doi:10.1177/22925503221146783)
Supplement: sj-docx-2-psg-10.1177_22925503221146783 - Supplemental material for Understanding the Appropriate and Beneficial Use of Before and After Photos in Breast Surgery: A North American Survey [file sj-docx-2-psg-10.1177_22925503221146783.docx]

**Supplement 2. Main Survey**

**Section 1:**

1. Have you had any aesthetic/cosmetic surgery?
   1. Yes
   2. Planning to undergo in <6 months
   3. Planning to undergo at some point in the future
   4. Considering undergoing
   5. No
2. If you answered "Yes", "Planning to undergo", or "Considering undergoing", please specify the specific surgery below (eg. facelift/rhytidectomy). If you answered "No", please type N/A.

______________________________________________

1. Have you had reconstructive surgery?
   1. Yes
   2. Planning to undergo in <6 months
   3. Planning to undergo at some point in the future
   4. Considering undergoing
   5. No
2. If you answered "Yes", "Planning to undergo", or "Considering undergoing", please specify the specific surgery below (eg. tendon repair). If you answered "No", please type N/A.

______________________________________________

1. Have you ever looked at another patient's before and after photographs similar to your surgery(ies) or potential surgery(ies)?
   1. Yes
   2. No
   3. Unsure
2. If you answered "Yes" or "No," please explain why or why not you chose to view before and after photographs in the space below.

______________________________________________

1. Did these images influence your decision with regards to consenting to surgery?
   1. Yes
   2. No
   3. Unsure
   4. N/A
2. If you answered "Yes" or "No," please provide detail as to how they impacted your decision in the space below. Please specifically address the positive or negative impact they had on your shared decision making.

______________________________________________

1. Did viewing the before and after photographs make you feel more confident consenting to surgery?
   1. Yes
   2. No
   3. Unsure
   4. N/A
2. Please specifically address whether you felt more empowered and/or more confident to consent after viewing the before and after photographs in the space below.

______________________________________________

1. What resources did you use prior to consenting to surgery? You may select all that apply.
   1. Physician expertise
   2. Text resources provided by health care practitioners
   3. Other text resources (not provided by health care practitioners)
   4. Advise from other patients who had similar conditions/surgeries
   5. Verbal testimonials through hospital resources (e.g. support groups/community events)
   6. Websites run by medical groups/organizations/governing bodies
   7. Websites run by individual physicians accredited by the Royal College of Physicians and Surgeons of Canada
   8. Websites run by other non-physicians
   9. Websites run by patient breast surgery communities
   10. Accredited plastic surgeon's social media
   11. Other patients'/influencers' social media
   12. Other: ____________________
2. Please provide specific details for all selected options above (eg. website name(s)). Please also indicate whether or not these resources included before and after photographs.

______________________________________________

**Section 2:**

SD = strongly disagree, D = disagree, NAND = neither agree nor disagree, A = agree, SA = strongly agree, N/A = not applicable

|  | **SD** | **D** | **NAND** | **A** | **SA** | **N/A** | **Comments** |
| --- | --- | --- | --- | --- | --- | --- | --- |
| 1. Before and after photographs are integral to the informed consent process |  |  |  |  |  |  |  |
| 2. Before and after photographs support the patient in contributing to the decision-making process |  |  |  |  |  |  |  |
| 3. I would recommend that patients look at before and after photographs prior to consenting to surgery |  |  |  |  |  |  |  |
| 4. Before and after photographs are an accurate representation of what to expect from surgery |  |  |  |  |  |  |  |
| 5. Physician-provided before and after photographs are widely available |  |  |  |  |  |  |  |
| 6. I believe there should be increased access to physician-provided before and after photographs |  |  |  |  |  |  |  |
| 7. Before and after photographs are widely available through "outside resources" |  |  |  |  |  |  |  |
| 8. I believe there should be increased access to before and after photographs from "outside resources" |  |  |  |  |  |  |  |
| 9. I believe physician-provided before and after photographs are more helpful than “outside resources” |  |  |  |  |  |  |  |
| 10. I believe "outside resources" are equally as helpful or more helpful than physician-provided before and after photographs |  |  |  |  |  |  |  |

Please share your comments on the selections you made above.

______________________________________________

**Section 3:**

SD = strongly disagree, D = disagree, NAND = neither agree nor disagree, A = agree, SA = strongly agree, N/A = not applicable

|  | **SD** | **D** | **NAND** | **A** | **SA** | **N/A** | **Comments** |
| --- | --- | --- | --- | --- | --- | --- | --- |
| **1. The intended photographic audience of before and after photographs are:** | | | | | | | |
| Office patients |  |  |  |  |  |  |  |
| Patients considering surgery |  |  |  |  |  |  |  |
| Patients’ families |  |  |  |  |  |  |  |
| The general public |  |  |  |  |  |  |  |
| General public excluding minors |  |  |  |  |  |  |  |
| **2. Appropriate places for patients to view before and after photographs are:** | | | | | | | |
| Anywhere |  |  |  |  |  |  |  |
| Anywhere on a secure site |  |  |  |  |  |  |  |
| Home |  |  |  |  |  |  |  |
| Doctor’s office |  |  |  |  |  |  |  |
| **3. Appropriate platforms to show patients before and after photographs are:** | | | | | | | |
| Websites |  |  |  |  |  |  |  |
| Social media |  |  |  |  |  |  |  |
| Private links sent to patients |  |  |  |  |  |  |  |
| Brochures/print media |  |  |  |  |  |  |  |
| Devices at the doctor's office |  |  |  |  |  |  |  |
| **4. Appropriate forms of presenting before and after photographs are:** | | | | | | | |
| Hand drawings |  |  |  |  |  |  |  |
| Formal illustrations |  |  |  |  |  |  |  |
| Unedited photographs |  |  |  |  |  |  |  |
| Edited photographs - lighting only (not photoshopped) |  |  |  |  |  |  |  |
| Videos |  |  |  |  |  |  |  |
| Simulations (3D/morphed) |  |  |  |  |  |  |  |
| In-person results from previous patients |  |  |  |  |  |  |  |
| **5. Standard clinical before and after photographs should include:** | | | | | | | |
| When the photographs were taken |  |  |  |  |  |  |  |
| Documentation required for informed consent |  |  |  |  |  |  |  |
| Testimonials |  |  |  |  |  |  |  |
| Consistent lighting (pre- and post-operative) |  |  |  |  |  |  |  |
| 1:1 size ratio between BAPs |  |  |  |  |  |  |  |
| Picture views: Front profile, side profile ± 45^o^ profile |  |  |  |  |  |  |  |
| Arms up view |  |  |  |  |  |  |  |
| Close-up of scars and deformities |  |  |  |  |  |  |  |
| Same body positions/standardized positions |  |  |  |  |  |  |  |
| Consistent patient exposure |  |  |  |  |  |  |  |
| Same backgrounds |  |  |  |  |  |  |  |
| Unaltered (no photoshop/filter) |  |  |  |  |  |  |  |
| More diversity (i.e. range of skin tones) |  |  |  |  |  |  |  |
| **6. Standard clinical before and after photographs should include pictures from:** | | | | | | | |
| During the surgery |  |  |  |  |  |  |  |
| Immediately after the surgery |  |  |  |  |  |  |  |
| 2 weeks after the surgery |  |  |  |  |  |  |  |
| 6 weeks after the surgery |  |  |  |  |  |  |  |
| 3 months after the surgery |  |  |  |  |  |  |  |
| 6 months after the surgery |  |  |  |  |  |  |  |
| 1 year after the surgery |  |  |  |  |  |  |  |
| **7. With regards to de-identifying patients, before and after photographs should have:** | | | | | | | |
| Identifiers fuzzed out/blacked out/covered |  |  |  |  |  |  |  |
| Identifiers erased/“photoshopped” out |  |  |  |  |  |  |  |
| No patient identifiers |  |  |  |  |  |  |  |
| All digital tags (name, location)/file name removed |  |  |  |  |  |  |  |
| **8. The following labels should be included in before and after photographs:** | | | | | | | |
| Age of patient |  |  |  |  |  |  |  |
| Time from surgery |  |  |  |  |  |  |  |
| All procedures performed (visible in photograph) |  |  |  |  |  |  |  |
| Any device, all device information (i.e. shape/size/position of implant) |  |  |  |  |  |  |  |
| Incision type |  |  |  |  |  |  |  |
| Diagnosis, previous treatment |  |  |  |  |  |  |  |
| **9. The range of outcomes that should be represented in before and after photographs should include:** | | | | | | | |
| Best results |  |  |  |  |  |  |  |
| Average results |  |  |  |  |  |  |  |
| Achievable/reproducible results |  |  |  |  |  |  |  |
| **10. Casual clothing, such as the following, should be included in before and after photographs:** | | | | | | | |
| Shirts without cleavage |  |  |  |  |  |  |  |
| Shirts with cleavage |  |  |  |  |  |  |  |
| Bathing suits |  |  |  |  |  |  |  |
| Patient photos need to be shown in casual clothing |  |  |  |  |  |  |  |
| **11. Fill in the blank: I believe a representative set of before and after photographs should include:** | | | | | | | |
| 3 photographs |  |  |  |  |  |  |  |
| 10 photographs |  |  |  |  |  |  |  |
| No limit on photographs |  |  |  |  |  |  |  |

Please share your comments on the selections you made above at the end of each question.

______________________________________________
